# Add-On Technologies That Aim to Improve Oocyte Quality and Embryo Implantation Potential

**DOI:** 10.3390/medicina61030367

**Published:** 2025-02-20

**Authors:** Nikos Petrogiannis, Maria Filippa, Kalliopi Chatzovoulou, Savvas Petrogiannis, Ioannis Filippas, Grigoris Grimbizis, Efstratios Kolibianakis, Katerina Chatzimeletiou

**Affiliations:** 1 ART Unit, Naval Hospital of Athens, 11521 Athens, Greece; np120@hotmail.com (N.P.); filippamarian@gmail.com (M.F.); s.petrog@hotmail.com (S.P.);; 2 Unit for Human Reproduction, 1st Department of Obstetrics and Gynaecology, Aristotle University Medical School, Papageorgiou General Hospital, 56403 Thessaloniki, Greece; grimbi@med.auth.gr (G.G.); stratis.kolibianakis@gmail.com (E.K.); katerinachatzime@hotmail.com (K.C.)

**Keywords:** platelet-rich plasma, in vitro maturation, artificial oocyte activation, preimplantation genetic testing, time-lapse imaging, mitochondrial replacement therapy, hyaluronan-rich culture media

## Abstract

Advancements in assisted reproductive technologies (ARTs) have led to the development of various add-on techniques aimed at improving oocyte quality and enhancing embryo implantation potential. These techniques target critical stages of both oocyte and embryo physiology, including oocyte growth and maturation, fertilization, chromosomal status, and embryo development. Key approaches involve the optimization of in vitro fertilization (IVF) protocols, recruiting capable follicles giving rise to dynamic oocytes to evolve, culture media supplementation, preimplantation genetic testing (PGT), and mitochondrial replacement therapy (MRT), all of which are designed to enhance oocyte competence through its function and metabolism. The use of PGT has been promising in selecting embryos suitable for transfer, thus optimizing implantation success. Emerging technologies, such as platelet-rich plasma treatment (PRP), time-lapse imaging (TLI), and hyaluronan-rich (HA) culture media, claim to improve ovarian rejuvenation and uterine receptivity, embryo selection, as well as embryo implantation potential, respectively. Evidence for certain add-on approaches remains limited, but ongoing research suggests that the use of such treatments may lead to increased clinical pregnancies and live birth rates, especially in poor-prognosis patients. The present review describes the current state of the add-on innovations, their mechanisms of action, as well as their possibilities to increase ART success rates.

## 1. Introduction

**Figure 1:**
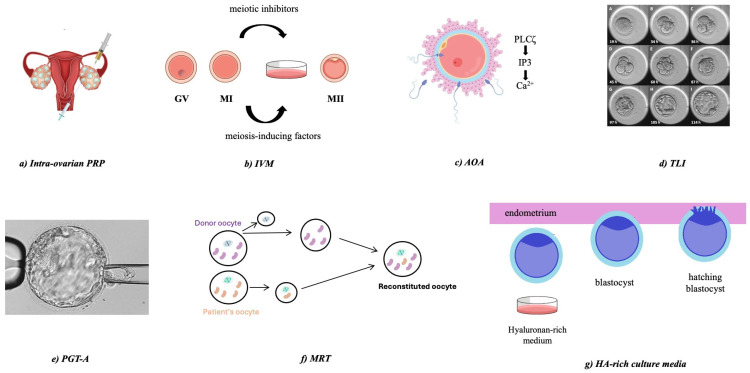
Schematic representation of the add-on techniques. Seven add-on techniques are discussed in the present review, namely: (**a**) intraovarian PRP treatment, (**b**) IVM, (**c**) AOA, (**d**) TLI, (**e**) PGT-A, (**f**) MRT, (**g**) HA-rich culture media. AOA: artificial oocyte activation; IVM: in vitro maturation; HA: hyaluronan; MRT: mitochondrial replacement therapy; PGT-A: preimplantation genetic testing for aneuploidies; PRP: platelet-rich plasma; TLI: time-lapse imaging.

Add-on techniques in ART are supplementary treatments designed to improve success rates in standard IVF and/or intracytoplasmic sperm injection (ICSI) cycles. Many of these techniques represent ways to enhance pregnancy outcomes, though their efficacy is often debated due to limited evidence. The current review presents some of the most common add-ons, targeting the oocyte and embryo enhancement, along with the evidence concerning their effectiveness and safety (Figure 1).

### 1.1. Platelet-Rich Plasma (PRP) Injection Treatment

PRP involves injecting platelet-rich plasma into the ovaries or uterus to rejuvenate tissue and potentially improve reproductive outcomes by stimulating the ovaries in order to induce follicle development and prompt good-quality oocytes for fertilization, particularly for women with poor ovarian reserve (POR) or thin endometrial lining. Ovarian PRP therapy utilizes a concentrated solution of growth factors and cytokines derived from a patient’s own blood [1]. PRP is produced by centrifuging whole blood, which typically consists of 55% plasma, 41% red blood cells, and 4% platelets and white blood cells [2]. During the process, red blood cells are removed, and the plasma becomes more highly concentrated with growth factors like transforming growth factor (TGF-β), vascular endothelial growth factor (VEGF), chemokines, and cytokines [3]. These components in PRP are thought to promote fertility by enhancing collagen synthesis, activating macrophages, stimulating angiogenesis, encouraging mitosis of endothelial cells, and supporting the recruitment of optimal oocytes for fertilization. To evaluate the effectiveness of PRP treatment, several diagnostic measures are employed. An increase in anti-Müllerian hormone (AMH) levels, along with a decrease in follicle-stimulating hormone (FSH) and luteinizing hormone (LH) levels, is expected and represents a good prognosis for patients following intraovarian PRP treatment, based on the most recent publications [1,4]. Additionally, ultrasound examinations are used to assess whether there is an increase in the antral follicle count (AFC), which further indicates a positive response to the therapy.

Promising evidence from meta-analyses suggests that autologous intraovarian PRP infusion may restore ovarian function by reactivating folliculogenesis and improving the hormonal profile. This treatment could potentially enable clinical pregnancy for selected groups of patients [5,6]. Specifically, intraovarian PRP injections have been associated with a statistically significant increase in serum anti-Müllerian hormone (AMH) levels [7]. Additionally, an increase in the average antral follicle count was observed in the same study following treatment. Interestingly, the outcomes of post-PRP intracytoplasmic sperm injection (ICSI) cycles showed improved success rates. Parameters such as the total number of retrieved oocytes, the number of mature oocytes, two-pronuclei zygotes, cleavage-stage embryos, and cancellation rates were all better in the post-PRP ICSI group compared to controls [5]. These findings highlight the potential of PRP in enhancing ovarian function and improving outcomes in assisted reproductive techniques.

Contrarily, both the effectiveness and benefits of the technique are debatable, with a large prospective randomized trial of intraovarian PRP showing no improvement in either mature oocyte yield or other parameters of IVF outcomes in patients less than 38 years old with POR [8]. According to the study, intraovarian PRP injection should not be applied in this population, as there is no clinical utility.

On the other hand, the use of intrauterine PRP injection in patients undergoing an ART cycle is showing encouraging results and existing data support its use on a clinical scale. Interestingly, based on a more recent study, the use of intrauterine treatment in patients with recurrent implantation failure (RIF) resulted in significantly higher clinical pregnancy and live birth rates, as well as lower miscarriage rates [9]. Similar results were also reproduced by others, thus recommending the use of intrauterine PRP in patients with a history of RIF [10,11], although more large and multicenter randomized controlled trials (RCTs) are needed to further validate its effect.

To conclude, based on the existing literature, PRP is still in experimental stages, with limited clinical data supporting its efficacy. While some studies seem promising in improving endometrial thickness or ovarian function, large-scale clinical trials are needed to confirm these findings. As such, PRP is not currently recommended as a standard treatment [12].

### 1.2. In Vitro Maturation of Oocytes (IVM)

IVM is an alternative technique to conventional IVF, involving the collection of immature cumulus–oocyte complexes (COCs) from antral follicles and their subsequent culture in the laboratory until they reach the metaphase II (MII) stage of meiosis [13,14,15]. This method differs from traditional controlled ovarian stimulation ART (COS-ART), as it requires little or no ovarian stimulation with or without in vivo hCG administration to induce ovulation. It is thus indicated for polycystic ovary syndrome (PCOS) patients or those who show intolerance to fertility drugs, like cancer patients. The IVM oocyte retrieval occurs earlier (between the 7th and 9th day of the cycle), whereas the COCs obtained should be from 8–12 mm follicles corresponding to early germinal vesicle (GV) stage to meiosis I (MI) oocytes within them. Once the COCs are matured in the laboratory, IVM oocytes undergo fertilization, usually by ICSI, and they are treated just like in vivo matured oocytes collected through usual COS-ART. It has been shown that in vitro matured oocytes can produce comparable results to those from standard ART protocols [16].

Four primary IVM protocols are commonly implemented in laboratory practice, namely: (1) the standard IVM protocol, including the collection of immature COCs that undergo IVM in a single step, (2) the biphasic IVM protocol, which is divided into two steps, with first the meiotic inhibition of the COCs at the GV stage and then inducing the final maturation through meiosis-inducing factors [17], (3) the “truncated” IVM protocol, comprising all types of oocytes (immature and mature) that are inseminated at different time points in the laboratory with duration from 4 to 36 h post-oocyte retrieval [18], and lastly (4) the “rescue IVM” or conventional IVM protocol, including the in vitro maturation of immature oocytes collected after a conventional IVF cycle, being inseminated 18–30 h post-retrieval [19].

As far as safety issues are concerned, the present available data do not support a globally negative impact of the use of IVM in clinical practice. More specifically, existing data indicate that IVM does not lead to an increased incidence of imprinting disorders, nor does it adversely affect the neonatal health and developmental outcomes of children conceived through this technique compared to those conceived via a conventional IVF protocol [20,21]. Additionally, aneuploidy rates appear comparable between these methods [22]. However, these findings are based on limited studies, and further research is necessary to confirm these conclusions. On the other hand, IVM represents a beneficial technique, requiring less time, reduced medical monitoring, and minimal or no hormone injections and blood tests. Cost-effectiveness analyses also highlight that IVM is a more affordable alternative compared to conventional ovarian stimulation protocols. Additionally, these characteristics have been associated with improved mental and psychological well-being of patients undergoing such procedures [23]. To pinpoint, successful implementation of IVM requires specialized expertise from both medical doctors and embryologists and appropriate patient selection fitting the right criteria. Furthermore, ongoing follow-up of children born through IVM is essential to ensure optimal clinical outcomes and long-term safety, although well-designed clinical trials per protocol are lacking in confidence.

### 1.3. Artificial Oocyte Activation (AOA)

During natural fertilization, phospholipase C zeta (PLCζ) enzyme, derived from sperm, activates the oocyte by triggering intracellular calcium (Ca^2+^) oscillations. These enable the meiosis completion of the oocyte and the decondensation of the sperm nucleus, thus leading to the formation of a zygote and the beginning of embryonic life. A deficiency in the intracellular calcium level, irrespective of sperm or oocyte origin, would lead to activation failure, even with the use of ICSI to perform fertilization. Human oocytes are tolerant to calcium level fluctuations to a critical threshold, so calcium levels can be increased artificially, i.e., AOA, by internal calcium stores, and/or external culture media, i.e., mechanical, electrical, or chemical stimuli that can generate a single calcium peak [24]. Mechanical AOA is performed by a slightly more invasive ICSI technique attempting to cause calcium release internally due to the extra injection pipette manipulations or by accumulating metabolically active mitochondria at the fertilization site [25]. Another approach using direct electric current creates pores in the oolemma, allowing influx of extracellular calcium [26], but its high degeneration rate and requirement of special equipment render chemical AOA the best choice.

AOA mimics calcium oscillations needed in cases of failed oocyte activation, which can occur due to sperm or oocyte factors. To stimulate chemical calcium release, AOA uses external sources, such as calcium ionophores, i.e., ionomycin or calcimycin. Calcimycin, known as A23187, an antibiotic, can bind bivalent ions (mainly Mn^2+^, Ca^2+^, and Mg^2+^), allowing their transport across cell membranes [24]. Ionomycin, having a higher potency due to its higher specificity for Ca^2+^ ions, is more widely used in ART, especially when combined with direct injection of 0.1 mol/L CaCl_2_, during ICSI [27].

Chemical AOA is effective for complete fertilization failure in previous IVF/ICSI cycles, low fertilization rates below 30%, and severe male factor infertility cases, i.e., globozoospermia [24]. Thus, injected oocytes are transferred immediately after ICSI to a pre-equilibrated ionophore solution for a 10–30 min culture, following washing. Moreover, usage of ionophores was adapted to increase embryo mitotic cleavage rate, in cases of previous embryonic arrest, developmental delay, or low blastocyst formation [28,29]. Since mitosis is also strongly Ca^2+^-dependent, such practices might be useful in clinical practice, as an add-on attempt.

According to the meta-analysis of 14 studies, chemical AOA increased live birth rates (LBRs) significantly in cases of recurrent ICSI fertilization failure, whereas success is patient-specific, highly indicated for low or failed fertilization in previous cycles, embryo developmental problems, or globozoospermia [30]. Nikiforaki and colleagues showed that sperm from a globozoospermic patient and AOA combined with ionomycin resulted in a better outcome during ICSI compared to calcimycin [27]. Interpreting AOA meta-analyses is controversial due to discrepancies between the variation in ionophore stimuli and concentration, exposure time, and number of exposures.

Ionophores do not seem to affect the oocyte cytoplasm, so they do not cause detectable effects on chromosomal segregation [31], gene expression, or embryo development [32]. Additionally, no increase in birth defects [33,34,35] or other developmental health issues in children aged 3–10 years born after AOA with ionophores has been reported [36]. In a recent AOA meta-analysis there were no significant differences in birth defects between the ICSI-AOA group and ICSI-only group, nor in the calcimycin or ionomycin subgroup [30].

As far as AOA safety is concerned, no increase in congenital defects has been reported, but its epigenetic risks, i.e., DNA methylation changes, require further research [37]. Potential epigenetic risks observed in experimental studies emphasize cautious application [38].

Though AOA can be beneficial in cases of failed fertilization after ICSI, its widespread use remains controversial. Most of the evidence supporting AOA comes from case studies or small trials, and large-scale RCTs are still needed to validate their routine use. Therefore, it is generally recommended for specific cases of fertilization failure, rather than as a universal approach for each patient without a history of fertilization failure.

### 1.4. Time-Lapse Imaging (TLI)

TLI represents a new incubation system that captures digital images of oocytes and/or embryos, during certain intervals, while being in culture. These images are collected to create a time-lapse video that demonstrates embryonic development, allowing for oocyte and embryonic quality assessment without removing oocytes and embryos from the incubator. Among the advantages of TLI is continuous monitoring of embryos without disturbing their environment. Furthermore, the analysis of morphokinetic parameters, such as the timing of cell divisions and the interval between cell stages, may improve embryo selection, potentially increasing implantation success rates. TLI represents a promising technology, although its clinical benefits still remain unclear. To start with, its effectiveness might be influenced by the variability of algorithms that are used for embryo development assessment, as well as culture conditions and the laboratory environment [39]. On the other hand, TLI might be very effective in other cases, such as research projects, protocol standardization, and better laboratory workflow, thus characterizing it as a non-add-on technology [40]. Interestingly, the latest Cochrane review on TLI concluded that there was no difference in clinical pregnancy, live birth, and/or ongoing pregnancy rates, miscarriages, and stillbirth between cases where TLI was used and those that were treated with conventional incubation methods [41]. Moreover, embryo selection through TLI softwares was not found to be more effective than the typical morphological assessment performed by the embryologists.

On the other hand, a more recent review analyzing the role of artificial intelligence (AI) in the analysis of oocytes in order to predict embryo developmental competence demonstrated the effectiveness of AI-based techniques during the implementation of IVF protocols. More specifically, an image of an oocyte was processed using a segmentation algorithm to identify specific regions of the oocyte, where the final features that were extracted were analyzed by a classification algorithm which was able to predict the maturation outcomes of the oocyte, thus increasing fertilization and implantation rates [42]. Although promising, several challenges need to be addressed before the universal use of AI-based technologies, such as the standardization of both imaging protocols and data formats, as well as the “ideal” training of machine learning models.

To conclude, while TLI and AI technologies may be convenient, providing continuous monitoring of oocytes and/or embryo development, as well as useful for research purposes and training of embryologists, its routine use as an add-on for all IVF patients is not yet justified, as based on the existing literature, it does not significantly improve LBRs.

### 1.5. Preimplantation Genetic Testing (PGT)

Human preimplantation embryos have been found to have an increased incidence of either meiotic or mitotic chromosomal abnormalities, namely aneuploidies. The proportion of cleavage-stage aneuploidies can go as high as 80%, being mainly attributed to advanced maternal age (AMA), whereas the blastocyst-stage aneuploidy rates are lower [43]. Thus, it was assumed that diagnosing pathological aneuploid embryos would be of benefit to the success rates of ART cycles.

PGT is a high-complexity procedure incorporating many specialized steps, namely ART through ICSI, embryo biopsy, cell tubing, traceability, cryopreservation, and genetic analysis to detect euploid embryos for transfer, and is used to screen embryos for genetic abnormalities before implantation, with PGT-A (A for aneuploidies) being the most prominent. Aneuploidy, a common cause of implantation failure and miscarriage, arises from chromosomal abnormalities in embryos. PGT-A enables the selection of euploid embryos, increasing the likelihood of successful implantation and reducing miscarriage risk.

PGT-A has long been the invasive diagnostic test of choice, former namely preimplantation genetic diagnosis (PGD), to obtain embryo genetic chromosomal information of euploidy or aneuploidy status, especially for AMA patients, over 40 years old, with the highest risk of embryonic meiotic abnormalities. It was later expanded to cases like RIF, recurrent pregnancy loss (RPL), and male infertility [44]. Genetic technology involved in PGT-A started with fluorescent in situ hybridization (FISH), being applied on a single blastomere from an eight-cell-stage embryo, to detect certain chromosomes [45]. Afterwards, PGT evolved with specific genetic testing techniques, like comprehensive chromosome screening (CCS), initially by array-comparative genomic hybridization (array-CGH), and now whole genome sequencing (WGS) and next-generation sequencing (NGS) [46], mainly on blastocyst biopsies [47,48]. Cleavage-stage biopsy markedly reduced embryonic reproductive potential. In contrast, trophectoderm biopsy had no measurable impact and may be used safely when embryo biopsy is indicated [49].

Current clinical practice PGT-A is an invasive, costly technique, taking place in high-complexity ART centers with proven good success rates [50]. Thus, it requires expertise and experience from the embryologists performing the biopsy, the tubing, and the cryopreservation, as well as modern genetic laboratories and skilled geneticists to perform and interpret the genetic results. The cost is usually imposed on the patient [51], and in certain patient cases PGT-A may decrease costs and time to a healthy pregnancy and live birth, like AMA women with many blastocysts, or those with RIF [52,53] and RPL [54], by avoiding hopeless embryo transfers (ETs) [55]. In 2019, the Preimplantation Genetic Diagnosis International Community (PGDIS) published a position statement, stating that PGT-A improved implantation, pregnancy, and live birth rates [56], which was rebutted later [57].

The efficacy and safety of PGT-A is a highly debatable issue among scientists arguing in favor and against its validity, whereas the majority of meta-analyses and evidence-based medicine demonstrate its usefulness in selected patient groups. In general it should be avoided and not recommended for everyone [12]. RCTs give inconsistent evidence of its effectiveness in younger women or those without a history of recurrent miscarriage [58]. Cornelisse and colleagues showed no increased LBRs after the first ET per woman randomized after PGT-A [59]. An RCT by Rubio and colleagues also failed to show higher LBRs [60]. The meta-analysis by Simopoulou and colleagues showed that PGT-A with comprehensive chromosomal screening (CCS) on day 3 or day 5 did not improve the general population clinical outcomes, but rather improved LBRs, strictly when performed on blastocyst-stage embryos of women over 35 years old [61]. The recent large Chinese RCT in young patients (20–37 years old) also failed to show improvement in LBRs per cycle [62]. Miscarriage rate and time to pregnancy should also be advocated as outcome measures, but results were controversial [59,63,64]. Studies from Verpoest, Rubio, and their teams found no significant difference in time to pregnancy between the PGT-A and control groups [60,63]. Delayed blastulation, poor blastocyst quality, maternal age over 38 years, obesity, previous RIF, and poor or multiple manipulations may reduce the LBR per euploid blastocyst transfer [65].

Another important issue to consider is the discrepancies in the diagnoses by different laboratories, i.e., the levels of mitotic mosaicism [66], raising concerns about the lack of standardization in the biopsy and genetic analysis techniques, leading to possible viable and healthy embryos being deselected or discarded due to false results. PGT-A of blastocysts, obstetrics, and follow-up of children born show no adverse effects [67], but there is a small risk of intrauterine growth restriction (IUGR) in many patient groups [68]. The Zheng et al., 2021 meta-analysis showed an increased risk of low birthweight, preterm delivery, pregnancy hypertensive disorders, and lower gestational age and birthweight in PGT pregnancies relative to spontaneously conceived pregnancies, mainly attributed to the freeze-all strategy [69]. The meta-analysis by Liang and colleagues demonstrated that PGT-A in RIF patients is associated with improved clinical outcomes, higher implantation rates (IRs), clinical pregnancy rates (CPRs), as well as LBRs [70]. The meta-analysis by Adamyan and colleagues showed that PGT-A improved the efficiency of ART, increasing both CPRs and LBRs, especially in women of AMA and with a poor prognosis [71]. PGT-A has technical limitations leading to false results, i.e., mosaic embryos (with both normal and abnormal cells), incorrectly classified as abnormal and not transferred or vice versa. Additionally, some chromosomal abnormalities, i.e., those within a small portion of the chromosome, may not be detected by PGT-A. Current invasive PGT-A is recommended for AMA, RIF, or RPL. Routine use in all IVF cycles is not advised due to inconsistent efficacy data and cost considerations [12].

The non-invasive PGT-A (ni-PGT) test would be of use and valid by performing genetic analysis either on blastocoel fluid [72] or spent culture media [73]. It is still developing and experimental, awaiting promising results for suitable clinical application [74]. The issues to be optimized concern its accuracy in the correspondence between the culture media genetic testing and the embryo inner cell mass, as well as contamination issues with parental genetic material [75,76]. Ni-PGT diagnostic accuracy has not yet been optimized to give safe results and is considered to have an experimental status, with its validation pending, although it will be the future method of choice, since no invasion and no cell removal is performed on the preimplantation embryo whose genetic status is being tested [77].

### 1.6. Mitochondrial Replacement Therapy (MRT)

ART techniques aiming to restore oocyte competence, also called “oocyte rejuvenation” methods, have been established [78], including cytoplasmic and mitochondrial transfer from healthy donors (i.e., healthy young females) to infertile patients (i.e., women with advanced maternal age). In particular, techniques of mitochondrial supplementation have been initially tested in mammals, showing encouraging results in ameliorating oocyte and embryonic quality [79,80]. Furthermore, trials have been reported in humans, leading to live births [81,82], but follow-up of the few children born is expected to estimate MRT’s safety.

In humans, cytoplasmic transfer has been performed by directly injecting a cytoplasmic fraction from the donor into the patient’s oocyte [82]. MRT techniques refer to the replacement of an impaired-quality cytoplasm with a more competent one, via the transfer of the nucleus into a recipient cytoplasm. Both techniques can be used for infertility treatments. In particular, options for patients carrying a mitochondrial disease or older AMA patients include: (1) the transfer of germinal vesicle (GV) from immature oocytes arrested at the meiosis I (MI) stage [83], (2) the transfer of metaphasic spindles at the meiosis II (MII) stage [84], (3) the transfer of pronuclei [85], and, eventually, (4) the transfer of first polar bodies (PB1s) [86], all originating from patient’s oocytes, which are subsequently transferred into the donor’s cytoplasm, from younger women with healthy mitochondria in the cytoplasm of their donor oocytes.

Although promising, attention should be drawn to the implementation of heterologous mitochondrial replacement techniques because of the unavoidable phenomenon of mitochondrial carryover arising from mitochondria adjacent to the nucleus, which might cause serious problems due to mtDNA heteroplasmy [87]. Even if it does not concern a mtDNA pathogenic variant, neutral heteroplasmy can indeed be the cause of serious impairments, as neutral heteroplasmic states have been associated with neurological disorders and developmental delay in mouse models [88,89]. In the most recent pilot study, the authors demonstrated the feasibility of MRT in patients with idiopathic infertility and repeated IVF failures. In fact, reconstructed oocytes successfully produced embryos that were able to implant, develop to full term, and result in seemingly healthy newborns or children, after the implementation of metaphasic spindles transfer (MST) [84]. However, it would be premature to conclude about the effectiveness of MST for infertility treatment due to the limitations of the study. Notably, mtDNA reversal was observed in one child born following MST, a finding that could have potential implications for MRT application. Such phenomena might have originated from the artificial connection of different-in-origin mtDNA and nuclear DNA, which can indeed have detrimental effects, all arising from the disruption of the mitochondrial–nuclear cross-talk.

Overall, while MRT techniques appear promising in treating female infertility related to ovarian aging, caution should be taken in their establishment in a clinical practice, as important issues, such as heteroplasmy, might adversely affect the progeny.

### 1.7. Hyaluronan (HA)-Rich Culture Media

HA is an essential natural macromolecule secreted by cumulus granulosa cells found in follicular and oviductal fluids, being shown to increase in the human uterus at the time of implantation [90,91]. HA is a straight chain, glycosaminoglycan polymer of the extracellular matrix composed of repeating units of the disaccharide [-D-glucuronic acid-β1,3-N-acetyl-D-glucosamine-β1,4-]n. Large hyaluronan polymers are space-filling, anti-angiogenic, and immunosuppressive and impede differentiation, possibly by suppressing cell–cell interactions or ligand access to cell surface receptors. Hyaluronan chains, which can reach 2 × 10^4^ kDa in size, are involved in ovulation, embryogenesis, protection of epithelial layer integrity, wound repair, and regeneration. Smaller polysaccharide fragments are inflammatory, immune-stimulatory, and angiogenic. They can also compete with larger hyaluronan polymers for receptors [92].

Except for being a promoter of cell-to-cell adhesion, HA, by producing a viscous medium, has been able to enhance embryo implantation, prohibiting its expulsion from the uterus [93]. HA with its autocrine and paracrine functions acts on CD44 receptors, vital for implantation. The HA primary CD44 receptor, being expressed on both the preimplantation embryo and the endometrial stroma, shows a peak at the most receptive time of embryo implantation [94]. Thus, HA regulates proliferation, differentiation, migration, and gene expression during endometrial decidualization and implantation, affecting even more normal embryo development [91].

Bovine studies showed HA improving embryo development by increasing the number of cells of blastocysts [91] by the CD44 activity and mitogen-activated protein kinase signaling. Thus, HA enhanced embryo quality [95], as well as improved frozen embryo viability maintenance, leading to increased implantation rates of thaw ETs [96,97]. As such, HA has been added to culture media (CM) during ET in ART, in either low or high commercially available concentrations. The proposed recipe is for the preimplantation embryo to be preincubated in the HA-enriched ET medium from 10 min to up to 4 h before ET.

In many cases, transferring a good-quality embryo onto a “fertile” endometrium with the correct lining and thickness may not be sufficient for its implantation, leading to failure of a promising ART attempt. For a successful pregnancy outcome, a chromosomally competent embryo is required, that is able to go through implantation by its apposition, adhesion, and invasion into the endometrium, via the necessary signaling factors involved. Several natural substances have been considered as sticky agents offering further support to the ET process, such as albumin, fibrin, collagen, and hyaluronan. There is no evidence to support these assumptions, since the in vivo embryo implantation status has not been unveiled yet, as well as the actual secretion occurring during this process.

There have been limited studies on the usage of albumin, fibrin, and collagen, for which there was no evidence to support an improvement in IRs or LBRs [98,99,100]. A recent Cochrane review by Heymann and colleagues compared two commercially available culture media, with no addition of HA, to either a low (0.125 mg/mL) or high (0.5 mg/mL) concentration [101]. Increased LBRs were found in the cohort with the high-HA constitution when compared to the low HA or no HA addition. The increase appeared for early cleavage-stage and blastocyst ETs, for good- and poor-prognosis patients. When evaluating the exposure time, in less than 10 min of HA bathing, no significant effect of the addition of high levels of HA was found. A more recent RCT by Yung and colleagues discovered no improvement in LBRs with 0.5 mg/mL HA compared to standard ET media [102]. Heymann and his team compared donor oocyte versus autologous oocyte cycles and concluded that, in donor cycles, HA addition showed little effect on LBRs and CPRs [103]. Three more studies performed only on frozen ET cycles also showed no evidence of improving the CPRs and LBRs.

Worries about implantation of suboptimal-quality embryos due to HA enrichment of ET media, leading to increased miscarriages, have not been indicated by any study. On the other hand, multiple CPRs were shown to be increased, probably due to the transfer of more than one embryo into an enriched HA ET medium.

To conclude, the addition of HA in ART ET media might increase LBRs after fresh transfers, without causing adverse effects, although this improvement was not evident in frozen ETs. There were multiple higher CPRs with HA-CM ET which should be further evaluated.

## 2. Discussion

Since the birth of the first IVF baby in 1978, the field of ART has undergone significant advancements thanks to many innovations. These developments have greatly enhanced the safety and effectiveness of treatments, providing substantial benefits to many individuals affected by infertility. However, the introduction of new techniques might be associated with interventions that have not been proven to be scientifically safe and suitable to improve the results of ART cases.

In this review we summarized several add-on techniques improving oocyte and preimplantation embryo quality, thus enhancing embryo implantation potential. Namely: PRP treatment, IVM of oocytes, AOA, TLI, PGT-A, MRT, and HA-rich culture media, as summarized in Table 1. Most of these techniques are currently used in clinical practice, although there are challenges that still need to be addressed before their safety and effectiveness are validated. In fact, many of the interventions discussed were not recommended for routine clinical practice for all infertility cases and therefore caution and scientific reasoning should be applied before their use (i.e., PRP). In certain cases, existing data have raised safety concerns or demonstrated a lack of efficiency (i.e., MRT). For other add-ons, insufficient evidence currently exists to justify their use in standard care (i.e., AOA). These should be further investigated through preclinical studies or within a clinical research framework, which requires ethics board approval, a well-designed and executed protocol, and commitment for long-term follow-up.

The current standard practice of evaluating cumulus cells, oocytes, and embryos in the ART lab is carried out through morphological parameters and morphokinetics with TLI and PGT, attempting to enhance their quality and potential by introducing the add-ons analyzed so far. Standard morphological assessment of oocytes and embryos is easily performed and without cost, but is subject to the variability of the human embryologist, whereas PGT has its invasive nature and flaws. With the constantly advancing technology via AI, robotics and automation, molecular biology, and genetics, there will soon be the usages of -omics biomarkers, i.e., proteomics, transcriptomics, metabolomics, and genomics, that non-invasively will enable with high accuracy, reproducibility, and safety the evaluation of the most dynamic ova to give rise to healthier offspring as quickly as possible. Proteomics will be able to discover specific protein biomarkers in either the follicular fluid or the culture media as well as the endometrium, showing the potential of oocyte and embryo development to lead to implantation and pregnancy. Secretomes, proteins, and metabolomics, i.e., amino acids, lipids, carbohydrates, and other developmental products identified in the spent culture media, may successfully profile the viability of oocytes and embryos. Genomics may be able to test and assess aneuploidy of the embryos through non-invasive ni-PGT-A by testing, through metabolomics of the embryo culture system, their genotype [104]. These -omics will enable embryologists to extract the corresponding information acquired through special culture techniques and technologies like mass spectrometry, gel electrophoresis, near-infrared spectroscopy (NIR), and fluorescence lifetime imaging (FLIM), without impairing the safety of the ova and embryos checked [105].

Moreover, another challenge in the IVF laboratory is the introduction of the artificial intelligence (AI) in the field of ART, aiming to enhance precision, outcomes, and decision-making processes. In fact, AI algorithms were generated in order to contribute to embryo selection, by analyzing TLI and morphological data to predict embryo viability and implantation potential with high accuracy [106]. The addition of such a software might indeed reduce human biases, while help improving success rates by identifying the “best” embryos for transfer among the ones having the potential to implant. AI algorithms are also able to evaluate sperm motility, morphology, and DNA integrity in order to optimize sperm selection processes for ICSI, thus ensuring better fertilization and developmental outcomes [107]. Likewise, AI may accelerate research projects and innovation by collecting data from laboratory routine use, providing great advancements in reproductive medicine. Although AI is a promising tool in the IVF laboratory, its integration requires witnessing, careful monitoring, and validation by clinicians and embryologists. A combination of AI and human expertise might improve both fertility outcomes and patient-specific treatments.

While add-on techniques raise both technical and ethical issues during their use in clinical practice, the field of ART is facing another major challenge, which is the introduction of artificial gametes in the IVF laboratory. More specifically, artificial gametes, derived from stem cells, hold the promise of enabling embryonic development. While artificial gametes are currently produced only from laboratory animals [108,109,110], significant progress is being made towards the development of artificial gametes from human cells [111]. Their availability from adult tissues could broaden reproductive aspects, allowing groups such as same-sex couples, post-menopausal women, or single people to conceive. However, the application of artificial gametes in clinical practice raises profound bioethical concerns, thus a bioethical framework needs to be established before the implementation of this approach.

It would be extremely beneficial to ART patients, researchers, embryologists, and clinicians to have access to and evaluate data and results of new treatments in national and international databases. This approach would enable continuous monitoring of their efficacy and safety by realizing their successes and their pitfalls or dangers, thus adopting, improving, or abandoning them accordingly. Innovation is the future, but evidence-based medicine is the key to lead the way with caution to validated science which will be of benefit in clinical practice [112]. Introducing add-ons to ART treatment should be considered very carefully, bearing in mind the patient’s medical history, especially their age, way of life, and cause of infertility. Patients with diminished ovarian reserve, autoimmune disorders, severe male factor infertility, or impaired lifestyle factors associated with higher risk of failing should be discouraged from trying add-ons. The purpose of introducing add-ons is to enhance success rates when there is a chance for it. Otherwise, it would be ethical and compassionate to advise ART patients on other ways of parenthood with less stress, risk, economic burden, and physical and emotional pain. For certain patient groups, specific add-ons might be of help to obtain their own biological offspring, whereas for many it may be futile and hopeless for it.

## 3. Conclusions

As far as modern technology and innovation are concerned, one should make a choice to use them when the appropriate evidence-based information is received to understand the risks and drawbacks entailed in the hope of achieving better fertility outcomes. Properly informed patients may have the free will to consent to add-ons, being aware of what to expect with emotional intelligence and honesty. Even health professionals would feel they are fulfilling their role of healing bodies and souls when a patient’s well-being is considered during the quest of obtaining a family. Overall, a balanced use of add-on techniques in clinical practice involves careful consideration of medical necessity, costs, and ethical implications. Taken all together, these add-ons contribute to advancing the field of ART, offering hope for improved outcomes, while highlighting the importance of personalized approaches in fertility treatments.

## Figures and Tables

**Table 1:** Add-on techniques categorized according to clinical procedures.

Timeline	Add-On Techniques
Pre-ART Treatment	PRP [1,2,3,4,5,6,7,8,9,10,11,12]
During Fertilization and Culture	IVM [13,14,15,16,17,18,19,20,21,22,23]
	AOA [24,25,26,27,28,29,30,31,32,33,34,35,36,37,38]
	TLI [39,40,41,42]
	MRT [78,79,80,81,82,83,84,85,86,87,88,89]
Before ET	PGT-A [12,43,44,45,46,47,48,49,50,51,52,53,54,55,56,57,58,59,60,61,62,63,64,65,66,67,68,69,70,71,72,73,74,75,76,77]
	HA-Rich Media [90,91,92,93,94,95,96,97,98,99,100,101,102,103]

Each add-on technique should be used when appropriate, during different clinical steps of the ART process. AOA: artificial oocyte activation; ART: assisted reproductive technology; ET: embryo transfer; IVM: in vitro maturation; HA: hyaluronan; MRT: mitochondrial replacement therapy; PGT-A: preimplantation genetic testing for aneuploidies; PRP: platelet-rich plasma; TLI: time-lapse imaging.

## Data Availability

No new data were created or analyzed in this study.
